# Origin of Polarization in Bismuth Sodium Titanate-Based Ceramics

**DOI:** 10.1021/jacs.3c13927

**Published:** 2024-02-14

**Authors:** Hangfeng Zhang, Marcin Krynski, A. Dominic Fortes, Theo Graves Saunders, Matteo Palma, Yang Hao, Franciszek Krok, Haixue Yan, Isaac Abrahams

**Affiliations:** † Department of Chemistry, Queen Mary University of London, Mile End Road, London E1 4NS, U.K.; ‡ School of Engineering and Materials Science, Queen Mary University of London, Mile End Road, London E1 4NS, U.K.; § Faculty of Physics, Warsaw University of Technology, Koszykowa 75, 00-662 Warszawa, Poland; ∥ STFC ISIS Facility, Rutherford Appleton Laboratory, Chilton Didcot, Oxfordshire OX11 OQX, U.K.; ⊥ School of Electronic Engineering and Computer Science, Queen Mary University of London, Mile End Road, London E1 4NS, U.K.

## Abstract

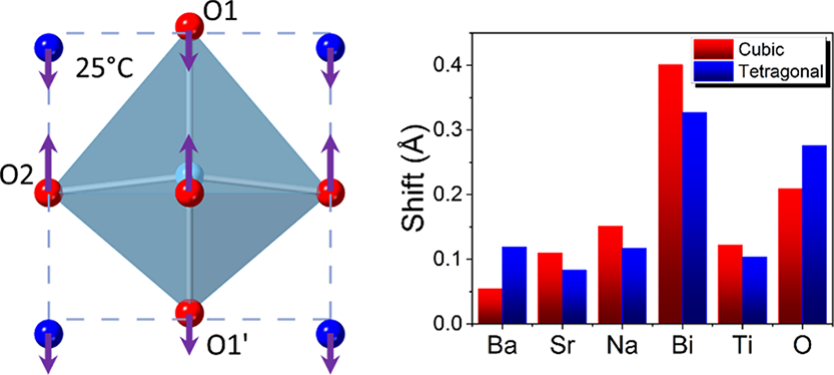

The classical view of the structural changes that occur at the ferroelectric transition in perovskite-structured systems, such as BaTiO_3_, is that polarization occurs due to the off-center displacement of the B-site cations. Here, we show that in the bismuth sodium titanate (BNT)-based composition 0.2(Ba_0.4_Sr_0.6_TiO_3_)–0.8(Bi_0.5_Na_0.5_TiO_3_), this model does not accurately describe the structural situation. Such BNT-based systems are of interest as lead-free alternatives to currently used materials in a variety of piezo-/ferroelectric applications. A combination of high-resolution powder neutron diffraction, impedance spectroscopy, and ab initio calculations reveals that Ti^4+^ contributes less than a third in magnitude to the overall polarization and that the displacements of the O^2–^ ions and the A-site cations, particularly Bi^3+^, are very significant. The detailed examination of the ferroelectric transition in this system offers insights applicable to the understanding of such transitions in other ferroelectric perovskites, particularly those containing lone pair elements.

## 1 Introduction

Ferroelectric (FE) materials find diverse applications, ranging from actuators,^1−4^ to energy storage capacitors,^5−7^ nonvolatile memory devices,^8−11^ and tunable communication devices,^12,13^ owing to their distinctive reversible polarization behavior under external electric fields. The establishment of the ferroelectric state involves electrical poling, which results in a parallel alignment of dipoles within the material. On removal of the electric field, only some of the polarization is lost, leaving a remnant polarization. The manifestation of ferroelectricity is highly sensitive to chemical composition, the presence of defects, and the electronic configuration of constituent atoms^14^ and arises from the competitive interplay between long-range Coulombic forces and short-range repulsions, which gives rise to FE and paraelectric (PE) phases, respectively.^15^ The classic example of a displacive ferroelectric is barium titanate, BaTiO_3_, which exhibits a FE to PE, transition at its Curie temperature, *T*_c_, of ca. 120 °C.^16^ This transition is associated with a change in crystallographic symmetry from polar tetragonal to nonpolar cubic. At the local level, the polar state has been described by an off-center displacement of Ti^4+^ ions with respect to the ideal centrosymmetric position within the B-site octahedra, which make up the framework of this perovskite-structured material. The noncentrosymmetric character of the tetragonal phase, in space group *P*4*mm*, means that refinement of its crystal structure requires the location of the origin to be fixed along the *c*-axis and thus the shifts of atoms in the structure are relative to the fixed atom position. Traditionally, the heavier atom, in this case, Ba, is chosen as the origin-defining atom.^17^ However, the overall polarization of a solid is due to the sum of all contributions from individual dipoles in the structure, some of which will be larger than others. The extent of the displacement of individual ions in a structure will depend on the electric susceptibility, which will be affected by their size, charge, and surroundings.

SrTiO_3_, often referred to as an incipient ferroelectric or quantum paraelectric, has a *T*_c_ value near 0 K and does not undergo a complete ferroelectric transition at any temperature due to the zero-point quantum fluctuation of the constituent ions preventing stable and long-range FE ordering.^18^ In comparison, BiFeO_3_ is ferroelectric with a rhombohedral perovskite structure at ambient conditions and transitions from a ferroelectric to a paraelectric phase at 825 °C.^19^ This ferroelectric character originates from the distortion of its simple cubic perovskite cell, involving both Fe–O bond length variations and octahedral rotations. Notably, Bi^3+^ ions exhibit a significant off-center displacement of 0.540 Å, contrasting the smaller 0.134 Å displacement of Fe^3+^ ions.^20^ In this work, we challenge the traditional view of the origin of polarization in perovskite FE materials through a detailed analysis of the atomic displacements in a lead-free relaxor FE system, 0.2(Ba_0.4_Sr_0.6_TiO_3_)–0.8(Bi_0.5_Na_0.5_TiO_3_) (BST246).

**Figure 1:**
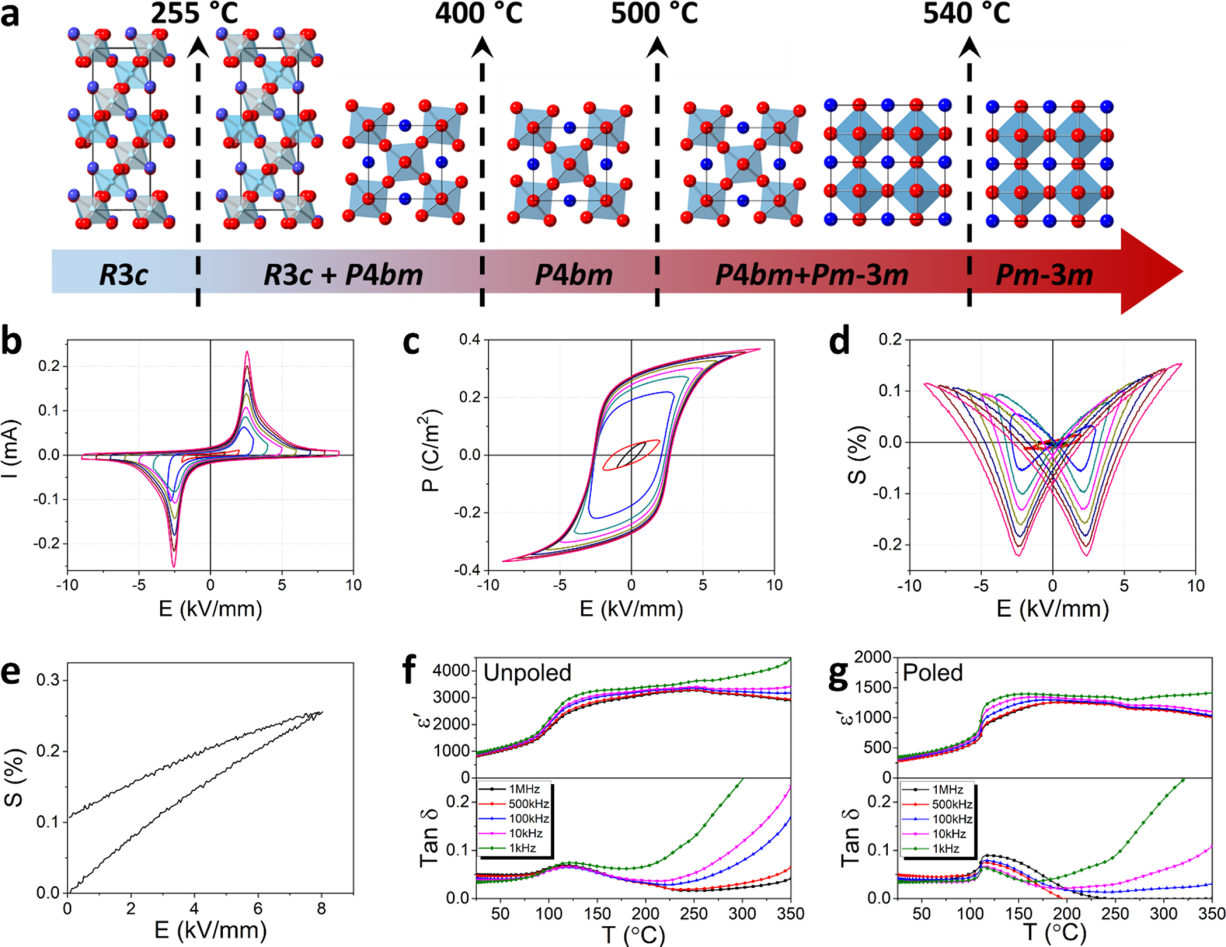
(a) Phase behavior of pure BNT on heating; (b) current density–electric field (*I*–*E*), (c) polarization–electric field (*P*–*E*), (d) bipolar strain–electric field (*S*–*E*), and (e) unipolar *S*–*E* loops for BST246 measured at room temperature; temperature dependencies of dielectric permittivity (ε′) and loss tangent (Tan δ) measured at selected frequencies for (f) unpoled and (g) poled BST246 ceramics.

The dominance of lead-based FE materials in commercial applications has raised concerns about their toxicity and environmental impact, leading to restrictions on their usage in electronic circuitry.^21^ Consequently, lead-free FE materials, particularly bismuth sodium titanate (Bi_0.5_Na_0.5_TiO_3_, BNT)-based systems, have attracted significant attention. As revealed by neutron diffraction studies,^22^ pure BNT exhibits complex phase behavior. These phases include a single rhombohedral phase observed below 255 °C, a mixture of rhombohedral and tetragonal phases in the range 255–400 °C, a single tetragonal phase in the range 400–500 °C, a combination of tetragonal and cubic phases from 500 to 540 °C, and finally, a single cubic phase above 540 °C (Figure 1a). These systems exhibit intriguing behavior characterized by nonergodic and ergodic states at temperatures below and above the depolarization temperature (*T*_d_), respectively.^23−25^ In the nonergodic state, the system can undergo an irreversible field-induced transition to the FE state as for a classical FE material, while the ergodic state is characterized by the reversibility of this transition. This so-called relaxor FE state exists up to the Burns temperature, *T*_B_, which corresponds to the transformation of the system to a complete paraelectric state. The origin of the relaxor FE state remains the subject of extensive research, with various theories proposed to explain the phenomenon.^26^ The most widely accepted theory is associated with the presence of polar nano regions (PNRs), characterized by easily switchable polar regions with random dipole orientations.

A-site substitution of Bi and/or Na in BNT significantly alters the phase behavior and dielectric properties. For example, Ba substitution enhances the piezoelectric response due to local structural heterogeneity associated with the coexistence of tetragonal and rhombohedral phases,^27−31^ while Sr substitution results in materials with high energy storage density attributed to field-induced phase transitions within the PNRs.^32^ The subtle structural changes associated with these substitutions are usually difficult to characterize using conventional X-ray powder diffraction techniques, not only due to their insensitivity to changes in the oxide ion sublattice but also due to the lack of resolution, compared to high-resolution neutron diffraction methods.

We have previously shown that Ba, Sr cosubstitution in the BNT-based system (Ba_0.4_Sr_0.6_TiO_3_)_*x*_(Bi_0.5_Na_0.5_TiO_3_)_1–*x*_ (0.5 ≤ *x* ≤ 1.0) leads to improved energy storage performance, attributed to field-induced phase transitions within the PNRs.^23^ In the present study, we explore the impact of a lower level (20 mol %) of Ba, Sr cosubstitution in BNT using high-resolution neutron diffraction, complemented by ab initio calculations and electrical measurements. As in our previous work, a Ba:Sr ratio of 2:3 is maintained because of the known low Curie point of the end member Ba_0.4_Sr_0.6_TiO_3_.^33^ While higher levels of Ba, Sr substitution are known to improve energy storage performance,^23^ lower levels of substitution are shown to enhance piezoelectric behavior.

The BST246 composition shows coexistence of tetragonal and cubic phases at room temperature, allowing for a direct comparison between the polar and nonpolar structures and their relative free energies. On heating, the gradual transformation from the polar tetragonal to nonpolar cubic states was monitored, allowing for the relative atomic displacements and their contribution to the overall polarization to be analyzed through this transition. Notably, this investigation reveals unexpected local structure evolution, challenging the traditional understanding of polarization behavior in perovskite-structured systems.

## 2 Experimental Section

### 2.1 Materials Synthesis

0.2(Ba_0.4_Sr_0.6_TiO_3_)–0.8(Bi_0.5_Na_0.5_TiO_3_) (BST246) was synthesized by a conventional solid-state route. First, powders of BNT and Ba_0.4_Sr_0.6_TiO_3_ (BST) were prepared separately using stoichiometric amounts of TiO_2_ (Aldrich, 99.8%) and either BaCO_3_ (Aldrich, 99%) and SrCO_3_ (Aldrich, 99.9%) or Bi_2_O_3_ (Aldrich, 99.9%) and Na_2_CO_3_ (Aldrich, 99.5%, preheated in an oven for 24 h at 200 °C) for BST and BNT, respectively. In each case, the starting compounds were ball-milled together in EtOH using a planetary ball mill with ZrO_2_ balls at 360 rpm for 4 h. After drying, the powders were calcined for 4 h at 840 or 1000 °C for BNT and BST, respectively. The resulting powders were mixed in the appropriate ratio and then ball-milled in EtOH for 4 h. After drying, the powder was pelletized at a pressure of 150 MPa, and the pellets sintered at 1150 °C for 3 h. Pellet densities were measured by the Archimedes method via the displacement of water.

### 2.2 Materials Characterization

For diffraction measurements, ceramic samples were crushed and ground into a fine powder. X-ray powder diffraction (XRD) was carried out on a PANAlytical X’Pert Pro diffractometer using Ni-filtered Cu–Kα radiation (λ = 1.5418 Å) at room temperature in flat plate θ/θ geometry over the 2θ range 5–120°, with a step width of 0.0334° and an effective count time of 50 s per step. Neutron diffraction data were collected on the high-resolution powder diffractometer HRPD at the ISIS Facility, Rutherford Appleton Laboratory, UK. Measurements were performed from room temperature up to 400 °C, with the sample contained in an 11 mm diameter vanadium can. Data sets corresponding to proton beam current equivalents of between 120 and 135 μA h were collected at room temperature, 150 and 400 °C, with shorter scans of around 35–60 μA h at other temperatures. Data acquired in the time-of-flight range 10–110 ms on the backscattering (158.46° < 2θ < 176.11°) detector bank, corresponding to *d*-spacings of 0.22 to 2.22 Å, were used in the subsequent analysis. The XRD data were fitted using a single phase model in the tetragonal space group *P*4*mm*.^34^ In the case of the higher resolution neutron diffraction data, a dual phase model consisting of cubic and tetragonal phases in space groups *Pm*-3*m*^23^ and *P*4*mm* was used. Rietveld refinement was carried out using the GSAS suite of programs.^35^ The morphology of ceramic cross sections was examined by scanning electron microscopy (SEM, FEI Inspect-F Oxford). Piezoresponse force microscopy (PFM) was carried out on a polished ceramic sample by using an atomic force microscope (Bruker Dimension Icon, US) with an SCM-PIT-V2 conductive probe (Bruker, US). A ceramic sample was initially ground down to approximately 150 μm in thickness and then subjected to ion milling until it became electron transparent. Transmission electron microscopy (TEM, JEM-F200, Japan) was employed to investigate the domain structure within the material.

### 2.3 Electrical Measurements

For electrical measurements, sintered pellets were ground to rectangular plates of approximate dimensions 4 × 4 × 0.3 mm^3^. Silver paste (Gwent Electronic Materials Ltd. Pontypool, UK.) electrodes were applied to both sides of the plates and heated at 500 °C. The temperature dependencies of dielectric permittivity and loss were measured using an LCR meter (Agilent 4284A) over the temperature range 25 to 400 °C at selected frequencies. Ferroelectric current density–electric field (*I–E*) and polarization–electric field (*P–E*) loops were measured with a ferroelectric tester (NPL, UK) with the applied electric voltage in a triangular waveform.^36^ The piezoelectric coefficient (*d*_33_) was measured using a quasi-static ZJ-3B *d*_33_ meter (Institute of Acoustics Academia Sinica, China).

### 2.4 Ab Initio Calculations

All ab initio calculations in this project were performed using the Vienna Ab initio Simulation Package (VASP).^37^ The Perdew–Burke–Ernzerhof^38^ functional with a cutoff value of 450 eV was applied as well as projector augmented wave potentials. The overestimation of electron delocalization was overcome by the application of the Strongly Constrained and Appropriately Normed functional (SCAN)^39^ together with the Dudarev^40^ approach of on-site Coulombic interaction with *U* = 2 eV for the d-orbitals of titanium atoms. All calculations were performed for 2 × 2 × 2 supercells containing 135 atoms. Ten atomic ensembles each, for the cubic and tetragonal phases, with different and random positions of cations and oxygen vacancies in each ensemble, were studied. In each configuration, 2 Ba, 3 Sr, 11 Bi, and 11 Na cations were randomly distributed on the A-site; similarly, 15 vacancies were randomly located on the 96 available oxide ion sites in the 2 × 2 × 2 supercell. All simulations were carried out with a global break condition for the electronic loop of 10^–7^ eV at the gamma point of the Brillouin zone. Free energies were calculated based on the normal modes obtained using the lattice dynamics method within the harmonic approximation.

## 3 Results and Discussion

At room temperature, BST246 shows typical ferroelectric polarization–electric field (*P*–*E*) hysteresis loops with only two current peaks in the first and third quadrants of the current density–electric field (*I–E*) loops (Figure 1b,c) up to a maximum field of 10 kV mm^–1^, attributable to domain switching. The coercive field and saturation polarization values are ca. 2.5 kV mm^–1^ and 3.8 C m^–2^, respectively. BST246 shows classical butterfly loops in its strain–electric field (*S–E*) plot using bipolar electric fields (Figure 1d). The value of the piezoelectric coefficient, *d*_33_, was 120 pC N^–^^1^ after poling (i.e., after the ferroelectric measurement). The unipolar *S*–*E* loop was measured under an electric field of 8 kV mm^–1^ (Figure 1e), and the *d*_33_* value (i.e., the *d*_33_ value under high field) was calculated to be 318 pm V^–1^.

*P–E*/*I–E* loops for BST246 at above-ambient temperatures are shown in Figure S1 up to a maximum field of 4 kV mm^–1^. In contrast to the two current peaks seen at room temperature, the data at 50 °C show four current peaks, labeled ±*E*_1_ and ±*E*_2_, in the first and third quadrants of the *I*–*E* loop with increased saturation polarization and decreased coercive field in the *P*–*E* loop. The observation of four current peaks in the *I*–*E* loops of BNT-based materials has previously been associated with a field-induced phase transition between a weak tetragonal polar phase and a strong polar rhombohedral phase.^41−43^ At room temperature, BST246 exhibits nonergodic relaxor behavior, where long-range order can be induced and maintained under an applied electric field. With increasing temperature, the activity of the polar regions increases and becomes more unstable, resulting in a lowering of the reverse electric field, *E*_1_. At 100 °C, the sample exhibits ergodic relaxor characteristics, where the long-range FE ordering is unstable, with four current peaks, one in each quadrant of the *I*–*E* loop and a double hysteresis seen in the *P*–*E* loop. This is consistent with the anomaly observed previously at *T*_d_ in the dielectric spectrum of BNT.^44^ At 125 °C, a complex *I*–*E* loop with two extra broad current peaks denoted as *E*_3_ and *E*_4_ is observed. The additional current peaks are suggested to be the result of field-induced phase transitions in the PNRs dispersed within the cubic phase. On further increase in temperature, the sample shows a narrow *P*–*E* hysteresis loop and four broad current peaks in the *I*–*E* loop. These four current peaks correspond to ±*E*_3_ and ±*E*_4_ seen at 125 °C. Although, as discussed below, the neutron diffraction data confirm a single cubic phase at 200 °C, it is suggested that some PNRs continue to exist in the material and are responsible for the observed relaxor-ferroelectric behavior at this temperature.

Figure 1f–g shows the variation of the dielectric permittivity and loss with temperature for unpoled and poled samples of BST246. At temperatures up to 100 °C, the dielectric permittivity exhibits less frequency dependency, and a dielectric loss peak is observed. At temperatures above 100 °C, the frequency dependence of dielectric permittivity becomes more pronounced up to 200 °C. On further increase in temperature, BST246 shows a significant increase in loss, which may be caused by the presence of oxygen vacancies.^45^ Such frequency dependence in BNT-based materials has been related to the activity and concentration of PNRs.^23^ At low temperatures, BST246 exhibits relatively large domain structures and shows frequency independent dielectric permittivity due to the stable domain wall configuration. As the temperature increases, the domain walls become more flexible and active, resulting in increased dielectric permittivity. With increasing temperature, polar nanoregions are formed and contribute to the dielectric permittivity. The frequency-dependent dielectric permittivity is a result of the varying activation energies and responses of the domains and PNRs, which result from their different sizes.^23^ However, heating also causes a decrease in the concentration of the polar tetragonal phase, leading to a decrease in dielectric permittivity. As a result of these two competing processes, the dielectric permittivity increases slowly and steadily over a wide temperature range. The permittivity value of unpoled BST246 is found to be higher than that of the poled sample. This is due to the poling process decreasing domain wall density.^46,47^ The sharp change in dielectric permittivity and loss at around 110 °C corresponds to *T*_d_, and it is a measure of the thermal instability of the domain structure.

**Figure 2:**
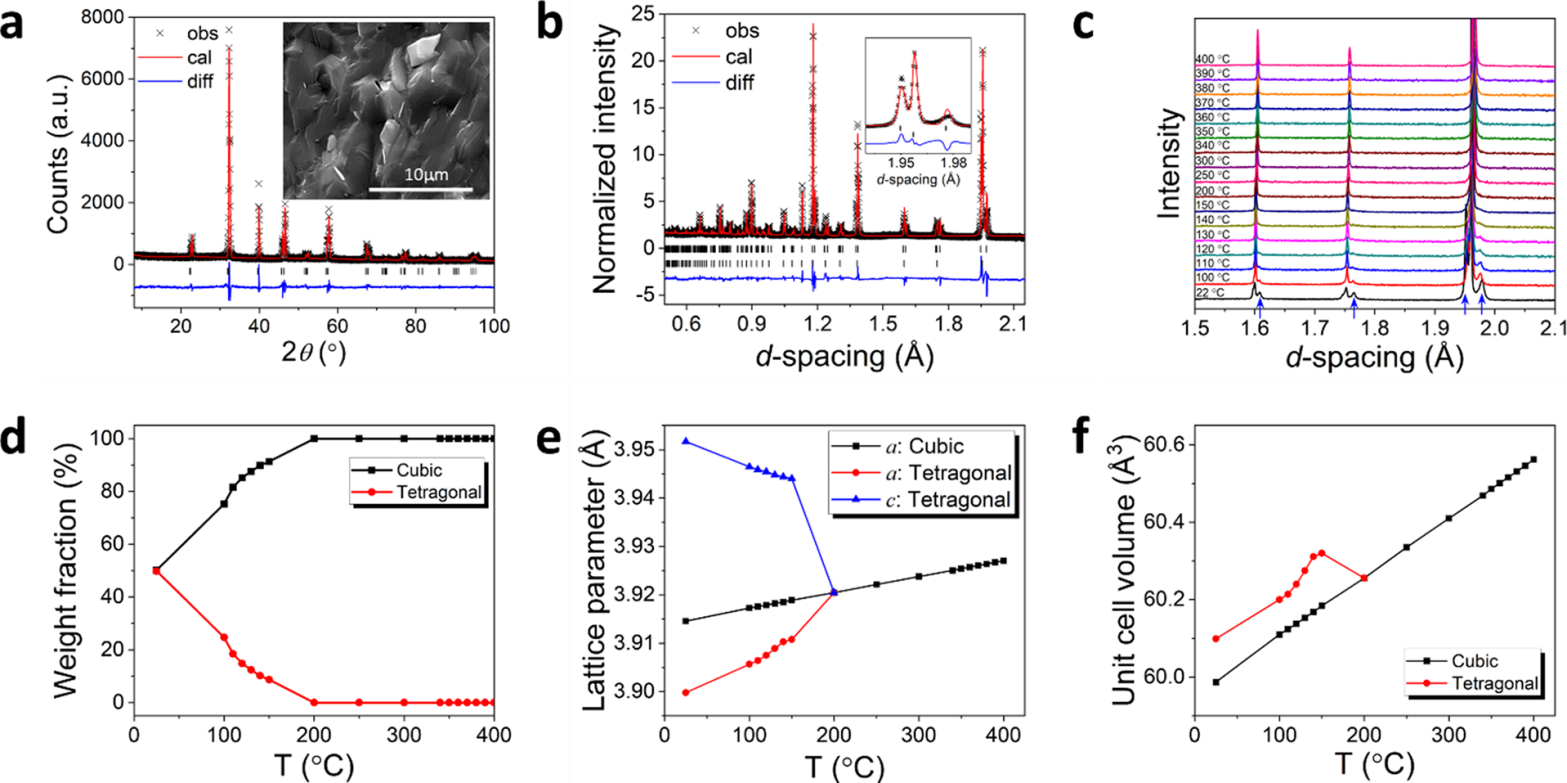
(a) XRD profile for BST246 fitted in space group *P*4*mm* with the SEM cross-sectional image inset; (b) neutron diffraction profile for BST246 fitted as a biphasic mixture of cubic (*Pm*-3*m*) and tetragonal (*P*4*mm*) phases with detail around 1.95 Å inset; thermal evolution of (c) neutron diffraction patterns, (d) phase weight fraction, (e) lattice parameters, and (f) unit cell volume for BST246.

The relative density of ceramic BST246 was found to be 95.2%, with SEM images showing a dense morphology and grain sizes of ca. 2 μm (Figure 2a inset). The XRD pattern of BST246 is well-fitted by a single phase model in space group *P*4*mm* (Figure 2a) consistent with previous studies of barium-doped BNT, which also exhibits a *P*4*mm* structure at barium concentrations above 11 mol %.^48^ Details of the neutron diffraction pattern for BST246 at room temperature (Figure 2b) reveal three diffraction peaks at *d*-spacings between 1.9 and 2.0 Å. Two of them correspond to the (200) and (002) planes in the tetragonal structure, but the third cannot be attributed to the tetragonal phase and must be associated with a secondary phase. The peaks of the secondary phase are readily indexed on a cubic cell, and therefore, the neutron data indicate that, at room temperature, BST246 is actually a mixture of tetragonal and cubic phases in space groups *P*4*mm* and *Pm*-3*m*, respectively. Rietveld refinement using a biphasic model revealed respective weight fractions of 49.86 and 50.14% at room temperature. Crystal and refinement parameters are given in Table S1 with the refined structural parameters and significant contact distances in Tables S2 and S3, respectively.

Neutron diffraction patterns for BST246 on heating to 400 °C are shown in Figure 2c. The peaks marked by arrows are associated with the tetragonal phase. On heating, the scattering intensity of the tetragonal phase peaks gradually decreases and disappears at temperatures above 150 °C. Figure 2d shows the thermal variation of the cubic and tetragonal phase weight fractions determined by Rietveld analysis and confirms that at 200 °C and above, BST246 is entirely cubic. The lattice parameter and unit cell volume of the cubic phase in the system linearly increase with temperature throughout the studied temperature range. This suggests that the tetragonal and cubic phases are identical in composition, and their coexistence at room temperature indicates that they possess similar free energies (Figure 2e,f).

**Figure 3:**
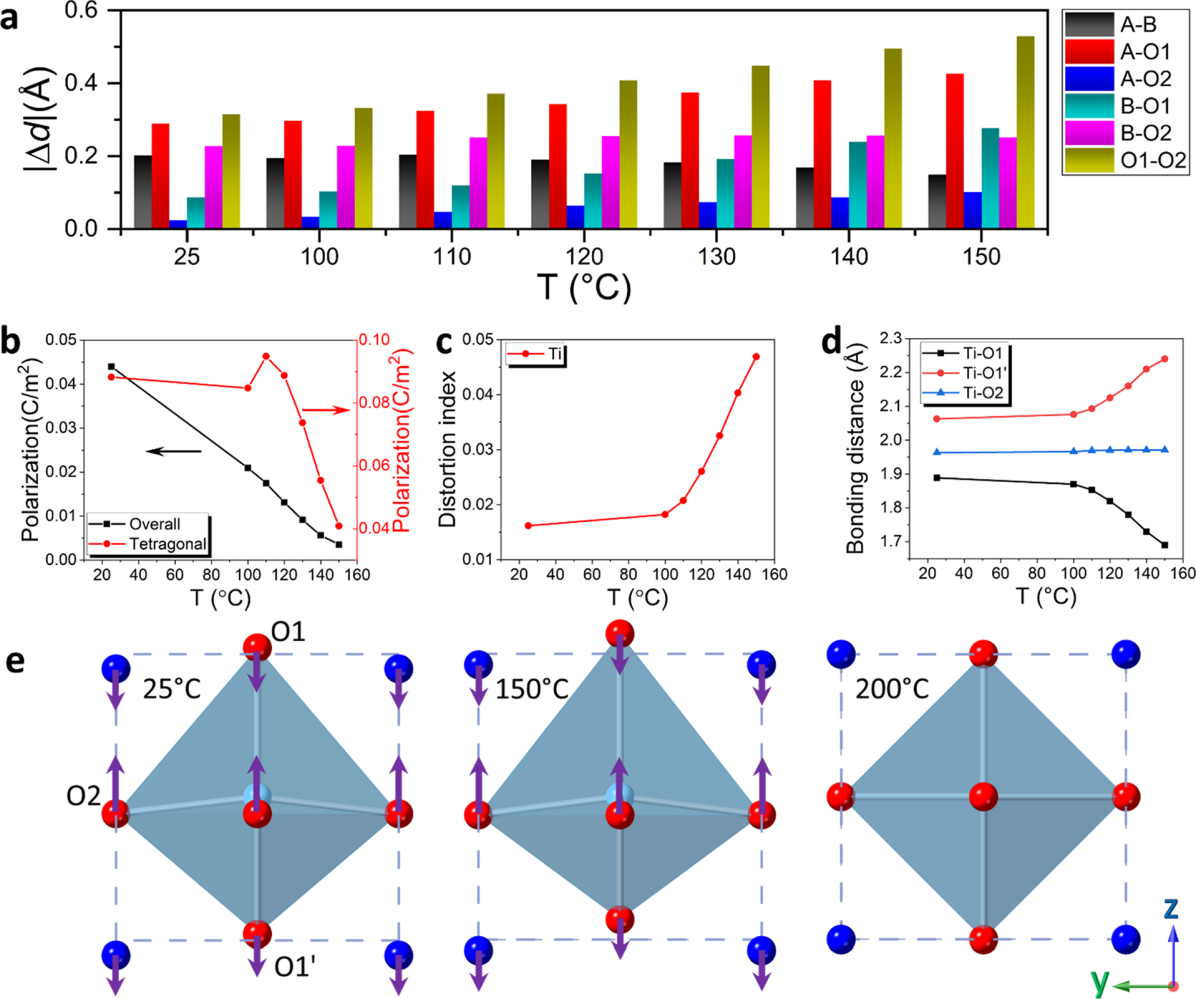
Thermal variation of (a) absolute relative atomic displacement, |Δ*d*|, with respect to the ideal centrosymmetric structure; temperature dependencies of (b) spontaneous polarization in the tetragonal phase of BST246 compared to the overall spontaneous polarization in the sample, (c) distortion index and (d) Ti–O bond distances in the tetragonal phase of BST246; (e) crystal structure of BST246 viewed in the *bc* plane, with atomic dipole moments parallel to the *c*-axis indicated by arrows. Blue, red, and cyan balls represent A-site (Ba/Sr/Bi/Sr) and Ti atoms, respectively.

In the tetragonal phase, the spontaneous polarization (*P*_s_) is associated with an overall dipole moment in the *c*-axis direction, which can be calculated by considering the displacement of individual atoms in the unit cell away from the ideal centrosymmetric case (eq 1)^49,50^:
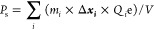
1where *m*_*i*_ is the number of atoms of type *i* in the unit cell, Δ*x*_*i*_ is the displacement away from the ideal centrosymmetric case. *Q*_*i*_e is the ionic charge and *V* is the unit cell volume. It should be noted here that as the position of Ti was fixed at (0.5, 0.5, 0.5) in the refinement, the calculated displacements and hence their contribution to the overall polarization are all relative to this reference point. It is therefore more useful to examine the relative atomic displacements between atom pairs. Figure 3a shows the absolute relative atomic displacements |Δ*d*| between the various atom pairs in the tetragonal phase over the studied temperature range. At room temperature, it is evident that the most significant relative displacement occurs between the O1 and O2 atoms and between the O1 and the A site cations. On increasing temperature, the displacements between the O1–O2 and A–O1 atom pairs become more significant compared to those between other atom pairs. Interestingly, the relative atomic displacement between the A and B site cations decreases with increasing temperature. This suggests that while the cations approach their centrosymmetric positions with increasing temperature, the oxide ions, particularly O1, move further away from their ideal positions leading to increasing distortion of the octahedral sites.

Figure 3b shows the thermal variation of spontaneous polarization in the tetragonal phase. There is a small decrease in spontaneous polarization on heating from room temperature to 100 °C, followed by a jump in polarization at 110 °C, which coincides with the *T*_d_ identified in the dielectric spectra, where the dipoles begin to show greater activity. Above this temperature, a general decrease in *P*_s_ is seen with increasing temperature until the disappearance of the tetragonal phase above 150 °C. Considering the weight fraction of the tetragonal phase in the sample, the overall polarization of the material shows a steady decrease with increasing temperature. The distortion of the titanate octahedra can be quantified using a bond length distortion index, *D:*(51)
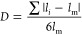
2where *l*_*i*_ and *l*_m_ are the bond length between Ti and the *i*th oxygen atom O_*i*_ and the mean Ti–O bond length, respectively. The thermal variation of *D* reflects that of the Ti–O1 distance shown in Figure 3c, with a small increase between room temperature and 100 °C, followed by a rapid increase from 100 to 150 °C.

The difference in the *a* and *c* tetragonal lattice parameters decreases with increasing temperature, resulting in a lower *c*/*a* ratio that is normally associated with a decrease in spontaneous polarization.^49^ One advantage of using high-resolution neutron diffraction to study the structural changes in this system is the sensitivity of this technique to small changes in the oxide ion sublattice, which enables accurate monitoring of the thermal evolution of the M–O (M = A/B-site cations) bond lengths in the system. The variation of Ti–O bond lengths in the tetragonal phase as a function of temperature is shown in Figure 3d, with values tabulated in Table S3. It is evident that while the Ti–O2 bond length (in the *a*–*b* plane) shows only a small increase with increasing temperature, the Ti–O1 bond lengths show a very significant change with increasing temperature above ca. 100 °C. Figure 3e shows the unit cell contents of the tetragonal phase compared to the ideal cubic structure, seen at 200 °C, with the relative atomic dipole moments contributing to the spontaneous polarization, indicated by arrows. At room temperature, the shift in the O1 positions leads to an off-center displacement in the *c*-axis of Ti within the B-site octahedron. This displacement increases with increasing temperature. In contrast, the relative displacement of the Ti atom with respect to the equatorial O2 atoms remains fairly constant with increasing temperature, as seen in Figure 3a. Interestingly, there is a slight increase in tetragonal cell volume between ca. 120 and 150 °C (Figure 2f), which may be interpreted as the lattice expanding somewhat to accommodate the increasing distortion of the B-site octahedra. At 200 °C, there is complete conversion of the sample to the cubic phase.

**Figure 4:**
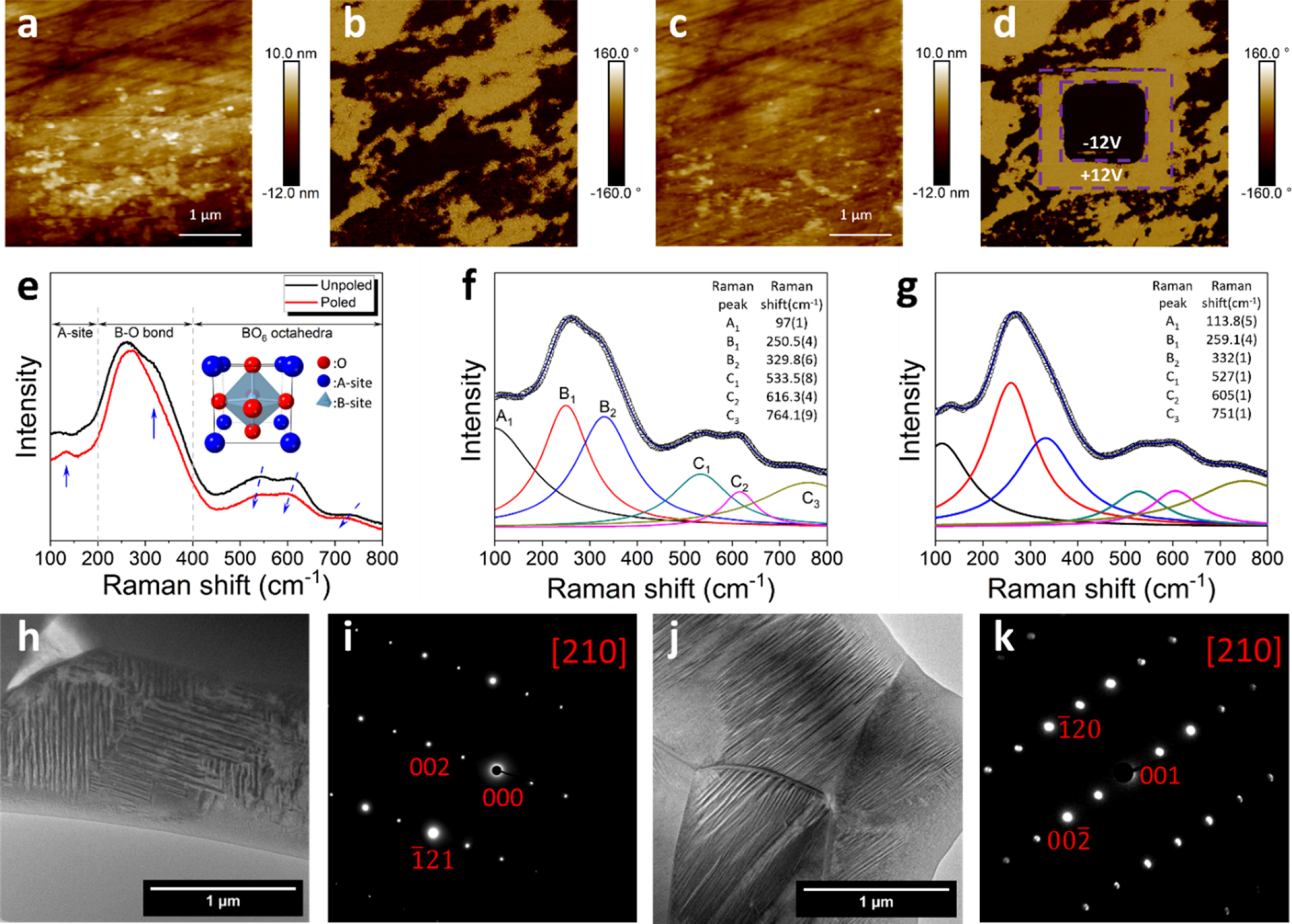
PFM images of (a, b) unpoled and (c, d) poled BST246 ceramic samples showing (a, c) topographic and (b, d) phase images; (e) Raman spectra of unpoled and poled BST246 ceramic samples, with fitted profiles for (f) unpoled and (g) poled samples; and TEM and SAED images of (h, i) unpoled and (j, k) poled BST246 samples.

PFM was carried out on a polished ceramic surface to study the domain structure of BST246. The morphology of the surface is shown in Figure 4a and exhibited a roughness of ca. 22 nm from the scanned 4 × 4 μm area. The domain structure is evident in the magnitude (Figure S2a) and phase (Figure 4b) images, showing areas of different contrasts. Figure 4c,d shows PFM images after application of ±12 V DC potentials to the surface through the PFM tip. The domain wall between two opposing domains, caused by the DC electric field, is apparent as a distinct square in both the phase (Figure 4e) and amplitude (Figure S2b) images. Electric ramping was performed at a single spot to reveal the amplitude and phase changes with applied field at 0.3 Hz (Figure S2c,d). A hysteresis loop was observed in the phase-electric field loop, showing domain switching behavior, with a coercive voltage of ca. 2 V corresponding to the valley point in the amplitude-electric field loop.

Raman spectroscopy was conducted on unpoled and poled BST246 ceramic samples to investigate electric field-induced changes in the local structure (Figure 4e). The obtained Raman spectra can be categorized into three distinct regions: Region A (≤200 cm^–1^), Region B (200–400 cm^–1^), and Region C (>400 cm^–1^), corresponding to the A-site, B–O bonding, and BO_6_ octahedral vibrational modes, respectively, and were deconvoluted into six Lorentzian peaks (Figure 4f,g). Compared to the spectrum of the unpoled sample, a notable intensification of the A_1_ peak and a weakening of the B_2_ peak are observed in the spectrum of the poled sample. The peak positions for A_1_, B_1_, and B_2_ modes shift toward a lower wavenumber, while those for C_1_, C_2_, and C_3_ modes shift toward a higher wavenumber after poling. The difference in peak positions (B_1_–B_2_) and (C_1_–C_2_) decreased after poling. These phenomena are attributed to the electric field-induced transition from nonergodic relaxor ferroelectric to ferroelectric states.^52,53^

BNT-based materials can exhibit various domain structures, including complex, nano, and lamellar structures corresponding to the FE state in *R*3*c*, relaxor antiferroelectric state in *P*4*bm*, and FE state in *P*4*mm* space groups, respectively.^7^ The TEM images of unpoled and poled BST246 ceramics reveal some large lamellar domain structures within the grains (Figure 4h,j), indicating the presence of the tetragonal (*P*4*mm*) structure, consistent with the neutron diffraction results. The observed domains in different grains are aligned and enlarged for poled BST246 as a result of electrical poling. This is confirmed by the selected area electron diffraction (SAED) images along the [210], [110], and [111], zone axes (Figures 4i and S3), where the diffraction spots can be entirely indexed in space group *P*4*mm*, with no additional superlattice diffraction spots observed. The diffraction spots of poled BST246 along the [210], [100], and [131] zone axes are also consistent with the *P*4*mm* structure (Figures 4k and S3).

**Figure 5:**
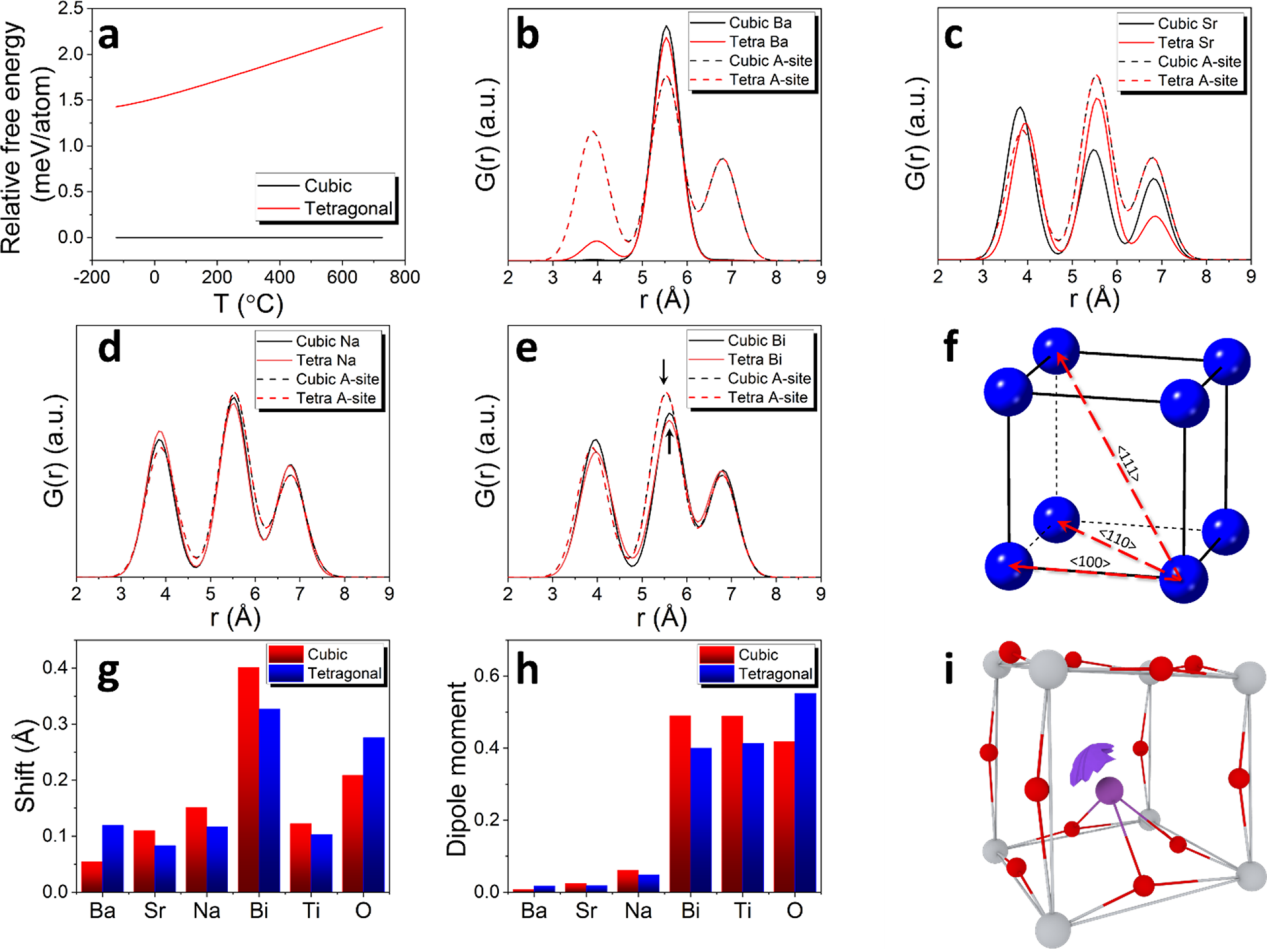
(a) Relative free energy per atom as a function of temperature based on ab initio simulations for the cubic and tetragonal phases; (b–e) radial density functions calculated for (b) Ba–Ba, (c) Sr–Sr, (d) Na–Na, and (e) Bi–Bi pairs (solid lines correspond to the free energy weighted distributions and dashed lines show patterns obtained for all A-site atoms); (f) schematic representations of distinct A-site atom alignments within the cubic/pseudocubic perovskite unit cell; (g) atomic shifts relative to the ideal centrosymmetric structure and (h) contributions to dipole moment calculated for individual atom types in the cubic and tetragonal phases of BST246 as derived from the relaxed ab initio models at room temperature; (i) schematic illustration showing bismuth coordination environment in the tetragonal phase derived from the relaxed ab initio model at room temperature showing the location of the Bi 6s^2^ lone pair, where purple, light gray, and red balls represent Bi^3+^, Ti^4+^, and O^2–^ ions, respectively.

The relative stability of the cubic and tetragonal phases was studied by calculating the Helmholtz free energy, the values of which were then used to calculate the occurrence frequency *w*_*i*_(*T*) of particular atomic ensembles in the macroscopic system where
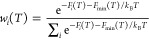
3*F*_*i*_(*T*) is the free energy of the *i*th atomic ensemble and *F*_min_(*T*) is the lowest observed value of the free energy among all the considered atomic ensembles. The value of *w*_*i*_(*T*_r_), where *T*_r_ is the sintering temperature (1150 °C), was then used in the calculation of the relative free energy difference between the cubic and tetragonal phase structures as shown in Figure 5a. It is visible that the cubic phase exhibits a lower free energy of around 1.5 meV/atom at −100 °C to around 2.5 meV/atom at around 700 °C. The relative free energy data indicate the possibility of phase coexistence at low temperatures, with increasing dominance of the cubic form with increasing temperature, as seen in the diffraction data.

The *w*_*i*_(*T*_r_) weights were also used to calculate mean radial distribution functions (Figure 5b–e), revealing the preferred distribution of cations compared to the average distribution of A-site cations (equivalent to a random distribution). The average A-site distributions for tetragonal and cubic phases are virtually indistinguishable, with three correlations visible at around 4, 5.6, and 6.9 Å for both cubic and tetragonal phases, corresponding to <100>, <110>, and <111> pairs in the ideal perovskite structure (Figure 5f). In the case of the Ba–Ba distribution, the <100> correlation is greatly diminished in the tetragonal phase with respect to the average A-site distribution, while the <111> correlation disappears completely. In the cubic phase, both these two correlations are absent, leaving only the <110> correlation present. The results indicate a strong preference for barium cations to maintain a significant separation from each other and not to be located in the nearest-neighboring A-site with respect to each other. In contrast, the Sr–Sr pair shows an increased preference for the location of neighboring Sr atoms in the <100> (and decreasing preference for Sr atoms in neighboring <110> and <111> A-sites) for both phases. The Bi–Bi and Na–Na distributions show little difference from the average distribution apart from a small shift in the <110> correlation to larger values in the Bi–Bi plot consistent with the observed displacement of this cation away from the center of the A-site due to the stereochemical activity of the Bi 6s^2^ lone pair.

Ionic displacements were calculated by comparing the atomic positions in the relaxed structures from the ab initio calculations to those in the ideal centrosymmetric structure. The obtained values were averaged between the considered atomic ensembles with *w*_*i*_(*T*_r_) weights. For cubic and tetragonal phases, the respective displacements (*d*) for A, B, and oxygen sites were found to be *d*_cubic_ = 0.24, 0.12, and 0.21 Å and *d*_tetragonal_ = 0.20, 0.10, and 0.28 Å. Interestingly, the displacements of individual types of A-site cations highlight that Bi exhibits significantly larger displacements than any of the other cation types (Figure 5g) in both tetragonal and cubic phases. The results indicate that most oxygen atoms are fairly strongly displaced compared to the ideal centrosymmetric sites, even more than most of the cation displacements (apart from Bi) in both phases. In the case of Ba^2+^, a relatively small displacement is seen in the cubic phase. The atomic contributions to the dipole moment for individual atom types based on the displacement data in Figure 5g are shown in Figure 5h, revealing significant large contributions to the dipole moment by Bi^3+^, Ti^4+^, and O^2–^ ions, contrasting with relatively small values for Ba^2+^, Sr^2+^, and Na^+^ in both phases. The overall dipole moment was calculated using the Berry phase approach with values for different atomistic ensembles also scaled using *w*_*i*_(*T*_r_) weights. The calculations show that the total (ionic plus electronic) weighted dipole moments are ***p*_cubic_** = (79.73, 281.02, 89.93) eÅ and ***p*_tetragonal_** = (289.96, 25.31, 573.14) eÅ.

## 4 Conclusions

BST246 ceramic was successfully synthesized by a solid-state reaction. High-resolution neutron diffraction and TEM analysis revealed two distinct phases, cubic and tetragonal, in the system, with roughly equal amounts present at room temperature. The piezoelectric coefficient values at low field, *d*_33_, and at high field, *d*_33_*, for poled BST246 were found to be 120 pC N^–^^1^ and 318 pm V^–1^, respectively. *I–E*/*P*–*E* results reveal nonergodic relaxor ferroelectric behavior from room temperature to 100 °C and ergodic relaxor ferroelectric behavior up to 200 °C. The depolarization temperature, *T*_d_, of BST246, is around 110 °C, which corresponds to a transition from nonergodic to ergodic states. Variable-temperature neutron diffraction studies reveal that the fraction of the tetragonal phase diminishes gradually and disappears above 150 °C. Despite this, BST246 still exhibits ergodic relaxor ferroelectric behavior at higher temperatures, suggesting the presence of polar nanoregions dispersed in the nonpolar cubic matrix.

Details of the structural changes that occur in the polar phase on heating reveal increasing distortion of the titanate octahedra despite decreases in *P*_s_. This somewhat counterintuitive behavior likely leads to a decrease in the stability of the tetragonal phase and its complete conversion to the cubic phase above 150 °C. The relative free energies of the tetragonal and cubic phases are consistent with their coexistence at lower temperatures with the cubic phase becoming increasingly more favored with increasing temperature. A nonrandom distribution of Ba^2+^ cations on the A-site is found, with short Ba–Ba contacts unfavored in both tetragonal and cubic phases. The absence of short Ba–Ba contacts is likely related to strain effects since the Ba^2+^ cation is significantly larger than the other A site cations (Ba^2+^: 1.42 Å, Sr^2+^: 1.26 Å, Na^+^: 1.18 Å, Bi^3+^: 1.17 Å for the ions in 8-coordinate geometry^54^).

Calculations of the atomic displacements and their contributions to the overall dipole moment within the two phases lead to some important conclusions. The cumulative dipole moments for A-site, B-site, and oxygen atoms exhibit comparable values, indicating nearly equal contributions to the spontaneous polarization. In particular, the Bi^3+^ cation exhibits a significant large displacement from the ideal centrosymmetric site associated with the Bi 6s^2^ lone pair of electrons and makes up a major part of the total A-site contribution to the dipole moment. Interestingly, while the Bi^3+^ cation is displaced toward one corner of the A-site, its lone pair resides close to the center of the A-site (Figure 5i). In contrast, the displacements of Ba^2+^, Sr^2+^, Na^+^, and Ti^4+^ are relatively small in both cubic and tetragonal phases. In the present case, despite the differences in displacement, the A-site, B-site, and oxygen atoms collectively contribute nearly equally to the overall dipole moment, with Ti^4+^ displacement accounting in magnitude for less than a third of the overall dipole moment. This challenges the conventional picture of the displacement of B-site cations within the octahedral sites as the primary cause of polarization. Whether this is generally true for other perovskite-structured ferroelectrics is unclear, and a similar analysis on other FE systems may be warranted. Comparing the present case to the classical BaTiO_3_ system, it should be noted that in this study, the displacement of the O^2–^ ions is significantly greater than that of both Ti^4+^ and Ba^2+^ ions, but when translated into contributions to the overall dipole moment, the contributions of Ti^4+^ and the O^2–^ ions are similar, while that for Ba^2+^ is small.

The in-depth examination of the tetragonal to cubic phase transition presented here, along with the associated variation in dielectric behavior within this A-site cosubstituted BNT system, holds promise for advancing our comprehension of such phase transitions in other ferroelectric perovskites, particularly those containing lone pair elements. Understanding the complex structural changes and polarization mechanisms in BST246 not only sheds light on the unique behavior of relaxor ferroelectrics but also provides crucial insights into tailoring and optimizing such materials for targeted applications. This comprehensive analysis of phase transitions and the dominance of specific atomic displacements in contributing to polarization challenges conventional theories, offering a new perspective for engineering novel compositions with enhanced functionalities for diverse technological advancements ranging from sensors and actuators to energy storage and beyond.

## Supporting Information Available

Variable temperature of PE/IE loops, PFM images, and details of XRD refined parameters and modification of modeling (PDF)

## References

[ref1] Zhang S.; Yu F. Piezoelectric Materials for High Temperature Sensors. J. Am. Ceram. Soc. 2011, 94 (10), 3153–3170. 10.1111/j.1551-2916.2011.04792.x.

[ref2] Setter N.; Damjanovic D.; Eng L.; Fox G.; Gevorgian S.; Hong S.; Kingon A.; Kohlstedt H.; Park N. Y.; Stephenson G. B.; Stolitchnov I.; Taganstev A. K.; Taylor D. V.; Yamada T.; Streiffer S. Ferroelectric Thin Films: Review of Materials, Properties, and Applications. J. Appl. Phys. 2006, 100 (5), 051606 10.1063/1.2336999.

[ref3] Pan W. Y.; Gu W. Y.; Taylor D. J.; Cross L. E. Large Piezoelectric Effect Induced by Direct Current Bias in PMN: PT Relaxor Ferroelectric Ceramics. Jpn. J. Appl. Phys. 1989, 28, 653–661. 10.1143/JJAP.28.653.

[ref4] Shvartsman V. V.; Lupascu D. C. Lead-Free Relaxor Ferroelectrics. J. Am. Ceram. Soc. 2012, 95 (1), 1–26. 10.1111/j.1551-2916.2011.04952.x.

[ref5] Meng N.; Ren X.; Santagiuliana G.; Ventura L.; Zhang H.; Wu J.; Yan H.; Reece M. J.; Bilotti E. Ultrahigh β-Phase Content Poly(Vinylidene Fluoride) with Relaxor-like Ferroelectricity for High Energy Density Capacitors. Nat. Commun. 2019, 10 (1), 4535 10.1038/s41467-019-12391-3. 31628311 PMC6800420

[ref6] Shimizu H.; Guo H.; Reyes-Lillo S. E.; Mizuno Y.; Rabe K. M.; Randall C. A. Lead-Free Antiferroelectric: XCaZrO 3 -(1 – x)NaNbO 3 System (0 ≤ *x* ≤ 0.10). Dalt. Trans. 2015, 44 (23), 10763–10772. 10.1039/C4DT03919J. 25700274

[ref7] Tan X.; Ma C.; Frederick J.; Beckman S.; Webber K. G. The Antiferroelectric ↔ Ferroelectric Phase Transition in Lead-Containing and Lead-Free Perovskite Ceramics. J. Am. Ceram. Soc. 2011, 94 (12), 4091–4107. 10.1111/j.1551-2916.2011.04917.x.

[ref8] Scott J. F. Status Report on Ferroelectric Memory Materials, Integr. Ferroelectr. 1998, 20 (February 2015), 15–23, 10.1080/10584589808238762.

[ref9] Jones R. E.; Zurcher P.; Chu P.; Taylor D. J.; Lii Y. T.; Jiang B.; Maniar P. D.; Gillespie S. J. Memory Applications Based on Ferroelectric and High-Permittivity Dielectric Thin Films. Microelectron. Eng. 1995, 29 (1–4), 3–10. 10.1016/0167-9317(95)00106-9.

[ref10] Fujimori Y.; Izumi N.; Nakamura T.; Kamisawa A. Sr 2 (Ta, Nb) 2 O 7 Ferroelectric Thin Film for Ferroelectric Memory FET. Integr. Ferroelectr. 1998, 21 (1–4), 73–82. 10.1080/10584589808202052.

[ref11] Yoshikazu Fujimori Y. F.; Naoki Izumi N. I.; Takashi Nakamura T. N.; Akira Kamisawa A. K. Application of Sr 2 Nb 2 O 7 Family Ferroelectric Films for Ferroelectric Memory Field Effect Transistor. Jpn. J. Appl. Phys. 1998, 37 (9S), 5207 10.1143/JJAP.37.5207.

[ref12] Zhang H.; Giddens H.; Yue Y.; Xu X.; Araullo-Peters V.; Koval V.; Palma M.; Abrahams I.; Yan H.; Hao Y. Polar Nano-Clusters in Nominally Paraelectric Ceramics Demonstrating High Microwave Tunability for Wireless Communication. J. Eur. Ceram. Soc. 2020, 40 (12), 3996–4003. 10.1016/j.jeurceramsoc.2020.04.015.

[ref13] Zhang H.; Gidden H.; Saunders T. G.; Liu N.; Aurallo-Peters V.; Xu X.; Palma M.; Reece M. J.; Abrahams I.; Yan H.; Hao Y. High Tunability and Low Loss in Layered Perovskite Dielectrics through Intrinsic Elimination of Oxygen Vacancies. Chem. Mater. 2020, 32 (23), 10120–10129. 10.1021/acs.chemmater.0c03569.

[ref14] Saito Y.; Takao H.; Tani T.; Nonoyama T.; Takatori K.; Homma T.; Nagaya T.; Nakamura M. Lead-Free Piezoceramics. Nature 2004, 432 (7013), 84–87. 10.1038/nature03028. 15516921

[ref15] Cohen R. E. Origin of Ferroelectricity in Perovskite Oxides. Nature 1992, 359, 136–138. 10.1038/358136a0.

[ref16] Haertling G. H. Ferroelectric Ceramics: History and Technology. In Ferroelectricity; Wiley-VCH Verlag GmbH: Weinheim, Germany, 2007; Vol. 818, pp 157–178.

[ref17] Buttner R. H.; Maslen E. N. Structural Parameters and Electron Difference Density in BaTiO3. Acta Crystallogr. Sect. B 1992, 48 (6), 764–769. 10.1107/S010876819200510X.

[ref18] Li X.; Qiu T.; Zhang J.; Baldini E.; Lu J.; Rappe A. M.; Nelson K. A. Terahertz Field–Induced Ferroelectricity in Quantum Paraelectric SrTiO3. Science (80-.) 2019, 364 (6445), 1079–1082. 10.1126/science.aaw4913. 31197011

[ref19] Catalan G.; Scott J. F. Physics and Applications of Bismuth Ferrite. Adv. Mater. 2009, 21 (24), 2463–2485. 10.1002/adma.200802849.

[ref20] Uwe H.; Sakudo T. Stress-Induced Ferroelectricity and Soft Phonon Modes in SrTiO3. Phys. Rev. B 1976, 13 (1), 271–286. 10.1103/PhysRevB.13.271.

[ref21] Rödel J.; Jo W.; Seifert K. T. P.; Anton E.; Granzow T.; Damjanovic D. Perspective on the Development of Lead-Free Piezoceramics. J. Am. Ceram. Soc. 2009, 92 (6), 1153–1177. 10.1111/j.1551-2916.2009.03061.x.

[ref22] Jones G. O.; Thomas P. A. Investigation of the Structure and Phase Transitions in the Novel A-Site Substituted Distorted Perovskite Compound Na 0.5 Bi 0.5 TiO 3. Acta Crystallogr. Sect. B Struct. Sci. 2002, 58 (2), 168–178. 10.1107/S0108768101020845. 11910154

[ref23] Zhang H.; Yang B.; Fortes A. D.; Yan H.; Abrahams I. Structure and Dielectric Properties of Double A-Site Doped Bismuth Sodium Titanate Relaxor Ferroelectrics for High Power Energy Storage Applications. J. Mater. Chem. A 2020, 8 (45), 23965–23973. 10.1039/D0TA07772K.

[ref24] Maurya D.; Zhou Y.; Wang Y.; Yan Y.; Li J.; Viehland D.; Priya S. Giant Strain with Ultra-Low Hysteresis and High Temperature Stability in Grain Oriented Lead-Free K0.5Bi0.5TiO3-BaTiO3-Na0.5Bi0.5TiO3 Piezoelectric Materials. Sci. Rep. 2015, 5 (1), 8595 10.1038/srep08595. 25716551 PMC4341219

[ref25] Zhou X.; Xue G.; Luo H.; Bowen C. R.; Zhang D. Phase Structure and Properties of Sodium Bismuth Titanate Lead-Free Piezoelectric Ceramics. Prog. Mater. Sci. 2021, 122 (July 2020), 100836, 10.1016/j.pmatsci.2021.100836.

[ref26] Bokov A. A.; Ye Z.-G. Recent Progress in Relaxor Ferroelectrics with Perovskite Structure. J. Mater. Sci. 2006, 41 (1), 31–52. 10.1007/s10853-005-5915-7.

[ref27] Chandrasekhar M.; Kumar P. Synthesis and Characterizations of BNT–BT and BNT–BT–KNN Ceramics for Actuator and Energy Storage Applications. Ceram. Int. 2015, 41 (4), 5574–5580. 10.1016/j.ceramint.2014.12.136.

[ref28] Ma C.; Tan X.; Dul’Kin E.; Roth M. Domain Structure-Dielectric Property Relationship in Lead-Free (1-x) (Bi1/2Na1/2) TiO3- x BaTiO3ceramics. J. Appl. Phys. 2010, 108 (10), 514093 10.1063/1.3514093.

[ref29] Tan X.; Aulbach E.; Jo W.; Granzow T.; Kling J.; Marsilius M.; Kleebe H.-J.; Rödel J. Effect of Uniaxial Stress on Ferroelectric Behavior of (Bi1/2Na1/2)TiO3-Based Lead-Free Piezoelectric Ceramics. J. Appl. Phys. 2009, 106 (4), 207827 10.1063/1.3207827.

[ref30] Bai W.; Li L.; Wang W.; Shen B.; Zhai J. Phase Diagram and Electrostrictive Effect in BNT-Based Ceramics. Solid State Commun. 2015, 206, 22–25. 10.1016/j.ssc.2015.01.004.

[ref31] Li F.; Lin D.; Chen Z.; Cheng Z.; Wang J.; Li C. C.; Xu Z.; Huang Q.; Liao X.; Chen L. Q.; Shrout T. R.; Zhang S. Ultrahigh Piezoelectricity in Ferroelectric Ceramics by Design. Nat. Mater. 2018, 17, 349 10.1038/s41563-018-0034-4. 29555999

[ref32] Wu J.; Mahajan A.; Riekehr L.; Zhang H.; Yang B.; Meng N.; Zhang Z.; Yan H. Perovskite Srx(Bi1–xNa0.97–xLi0.03)0.5TiO3 Ceramics with Polar Nano Regions for High Power Energy Storage. Nano Energy 2018, 50 (June), 723–732. 10.1016/j.nanoen.2018.06.016.

[ref33] Zhou L.; Vilarinho P. M.; Baptista J. L. Dependence of the Structural and Dielectric Properties of Ba 1- x Sr x TiO 3 Ceramic Solid Solutions on Raw Material Processing. J. Eur. Ceram. Soc. 1999, 19, 2015–2020. 10.1016/S0955-2219(99)00010-2.

[ref34] Jones G. O.; Kreisel J.; Thomas P. A. A Structural Study of the (Na 1– x K x) 0.5 Bi 0.5 TiO 3 Perovskite Series as a Function of Substitution (x) and Temperature. Powder Diffr. 2002, 17 (4), 301–319. 10.1154/1.1505047.

[ref35] Larson A. C.; Dreele R. B. Los Alamos National Laboratory Report, 1987.

[ref36] Viola G.; Saunders T.; Wei X.; Chong K. B.; Luo H.; Reece M. J.; Yan H. Contribution of Piezoelectric Effect, Electrostriction and Ferroelectric/Ferroelastic Switching to Strain-Electric Field Response of Dielectrics. J. Adv. Dielectr. 2013, 03 (01), 1350007 10.1142/S2010135X13500070.

[ref37] Kresse G.; Hafner J. Ab Initio Molecular Dynamics for Liquid Metals. Phys. Rev. B 1993, 47 (1), 558–561. 10.1103/PhysRevB.47.558. 10004490

[ref38] Perdew J. P.; Burke K.; Ernzerhof M. Generalized Gradient Approximation Made Simple. Phys. Rev. Lett. 1996, 77 (18), 3865–3868. 10.1103/PhysRevLett.77.3865. 10062328

[ref39] Furness J. W.; Kaplan A. D.; Ning J.; Perdew J. P.; Sun J. Accurate and Numerically Efficient R2SCAN Meta-Generalized Gradient Approximation. J. Phys. Chem. Lett. 2020, 11 (19), 8208–8215. 10.1021/acs.jpclett.0c02405. 32876454

[ref40] Dudarev S.; Botton G. Electron-Energy-Loss Spectra and the Structural Stability of Nickel Oxide: An LSDA+U Study. Phys. Rev. B: Condens. Matter Mater. Phys. 1998, 57 (3), 1505–1509. 10.1103/PhysRevB.57.1505.

[ref41] Ma C.; Guo H.; Beckman S. P.; Tan X. Creation and Destruction of Morphotropic Phase Boundaries through Electrical Poling: A Case Study of Lead-Free (Bi 1/2 Na 1/2) TiO 3 – BaTiO 3 Piezoelectrics. Phys. Rev. Lett. 2012, 109 (10), 107602 10.1103/PhysRevLett.109.107602. 23005327

[ref42] Mahajan A.; Zhang H.; Wu J.; Ramana E. V.; Reece M. J.; Yan H. Effect of Phase Transitions on Thermal Depoling in Lead-Free 0.94(Bi 0.5 Na 0.5 TiO 3)–0.06(BaTiO 3) Based Piezoelectrics. J. Phys. Chem. C 2017, 121 (10), 5709–5718. 10.1021/acs.jpcc.6b12501.

[ref43] Viola G.; MKinnon R.; Koval V.; Adomkevicius A.; Dunn S.; Yan H. Lithium-Induced Phase Transitions in Lead-Free Bi 0.5 Na 0.5 TiO 3 Based Ceramics. J. Phys. Chem. C 2014, 118 (16), 8564–8570. 10.1021/jp500609h.

[ref44] Schütz D.; Deluca M.; Krauss W.; Feteira A.; Jackson T.; Reichmann K. Lone-Pair-Induced Covalency as the Cause of Temperature- and Field-Induced Instabilities in Bismuth Sodium Titanate. Adv. Funct. Mater. 2012, 22 (11), 2285–2294. 10.1002/adfm.201102758.

[ref45] Kang B. S.; Choi S. K.; Park C. H. Diffuse Dielectric Anomaly in Perovskite-Type Ferroelectric Oxides in the Temperature Range of 400–700 °C. J. Appl. Phys. 2003, 94 (3), 1904–1911. 10.1063/1.1589595.

[ref46] Tan Y.; Zhang J.; Wu Y.; Wang C.; Koval V.; Shi B.; Ye H.; McKinnon R.; Viola G.; Yan H. Unfolding Grain Size Effects in Barium Titanate Ferroelectric Ceramics. Sci. Rep. 2015, 5, 9953 10.1038/srep09953. 25951408 PMC4423446

[ref47] Zhang M.; Chen Z.; Yue Y.; Chen T.; Yan Z.; Jiang Q.; Yang B.; Eriksson M.; Tang J.; Zhang D.; Shen Z.; Abrahams I.; Yan H. Terahertz Reading of Ferroelectric Domain Wall Dielectric Switching. ACS Appl. Mater. Interfaces 2021, 13 (10), 12622–12628. 10.1021/acsami.1c00523. 33685119

[ref48] Jo W.; Daniels J. E.; Jones J. L.; Tan X.; Thomas P. A.; Damjanovic D.; Rödel J. Evolving Morphotropic Phase Boundary in Lead-Free (Bi[Sub 1/2]Na[Sub 1/2])TiO[Sub 3]–BaTiO[Sub 3] Piezoceramics. J. Appl. Phys. 2011, 109 (1), 014110 10.1063/1.3530737.

[ref49] Shimakawa Y.; Kubo Y.; Nakagawa Y.; Goto S.; Kamiyama T.; Asano H.; Izumi F. Crystal Structure and Ferroelectric Properties of ABi2Ta2O9(A=Ca, Sr, and Ba). Phys. Rev. B 2000, 61 (10), 6559–6564. 10.1103/PhysRevB.61.6559.

[ref50] Yan H.; Zhang H.; Ubic R.; Reece M. J.; Liu J.; Shen Z.; Zhang Z. A Lead-Free High-Curie-Point Ferroelectric Ceramic, CaBi2Nb 2O9. Adv. Mater. 2005, 17 (10), 1261–1265. 10.1002/adma.200401860.

[ref51] Baur W. H. The Geometry of Polyhedral Distortions. Predictive Relationships for the Phosphate Group. Acta Crystallogr. Sect. B Struct. Crystallogr. Cryst. Chem. 1974, 30 (5), 1195–1215. 10.1107/S0567740874004560.

[ref52] Liu X.; Zhai J.; Shen B. Local Phenomena in Bismuth Sodium Titanate Perovskite Studied by Raman Spectroscopy. J. Am. Ceram. Soc. 2018, 101 (12), 5604–5614. 10.1111/jace.15875.

[ref53] Huangfu G.; Zeng K.; Wang B.; Wang J.; Fu Z.; Xu F.; Zhang S.; Luo H.; Viehland D.; Guo Y. Giant Electric Field–Induced Strain in Lead-Free Piezoceramics. Science (80-.). 2022, 378 (6624), 1125–1130. 10.1126/science.ade2964. 36480626

[ref54] Shannon R. D. Revised Effective Ionic Radii and Systematic Studies of Interatomic Distances in Halides and Chalcogenides. Acta Crystallogr., Sect. A 1976, 32 (5), 751–767. 10.1107/S0567739476001551.

