# Supplementary material for: Origin of Polarization in Bismuth Sodium Titanate-Based Ceramics
Source: J Am Chem Soc. 2024 Feb 14;146(8):5569–79. doi: 10.1021/jacs.3c13927 (PMC10910510; doi:10.1021/jacs.3c13927)
Supplement: Supplementary file 1 — ja3c13927_si_001.pdf [file ja3c13927_si_001.pdf]

Supporting information

Origin of polarization in bismuth sodium titanate based ceramics

Hangfeng Zhang,[1,2] Marcin Krynski,[3] A. Dominic Fortes,[4] Theo Graves Saunders,[2] Matteo Palma, [1] Yang Hao,[5] Franciszek Krok,[3] Haixue Yan,[2*] Isaac Abrahams[1*]

[1]Department of Chemistry, Queen Mary University of London, Mile End Road, London E1 4NS, UK.

[2]School of Engineering and Materials Science, Queen Mary University of London, Mile End Road, London E1 4NS, UK.

[3]Faculty of Physics, Warsaw University of Technology, Koszykowa 75, 00-662 Warszawa, Poland.

[4]STFC ISIS Facility, Rutherford Appleton Laboratory, Chilton Didcot, Oxfordshire, OX11 OQX, UK.

[5]School of Electronic Engineering and Computer Science, Queen Mary University of London, Mile End Road, London E1 4NS, UK.

Corresponding Authors

H. Yan	tel: +44 207 882 5164	email: h.x.yan@qmul.ac.uk
I. Abrahams	tel: +44 207 882 3235	email: i.abrahams@qmul.ac.uk

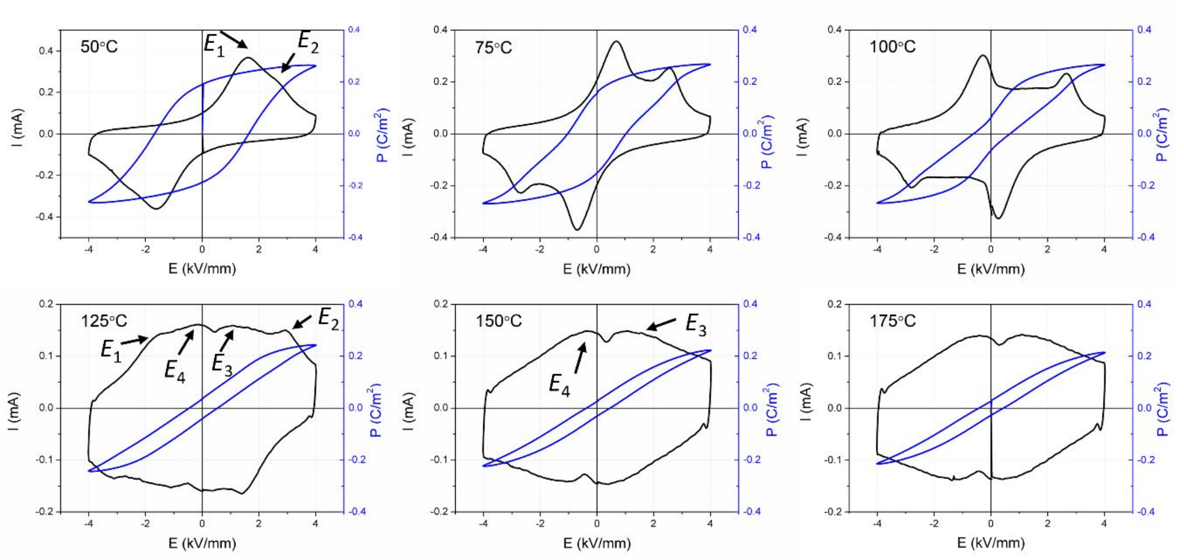

Figure S1. Current-electric field (I-E) and polarization-electric field (P-E) loops for BST246 measured at selected temperatures.

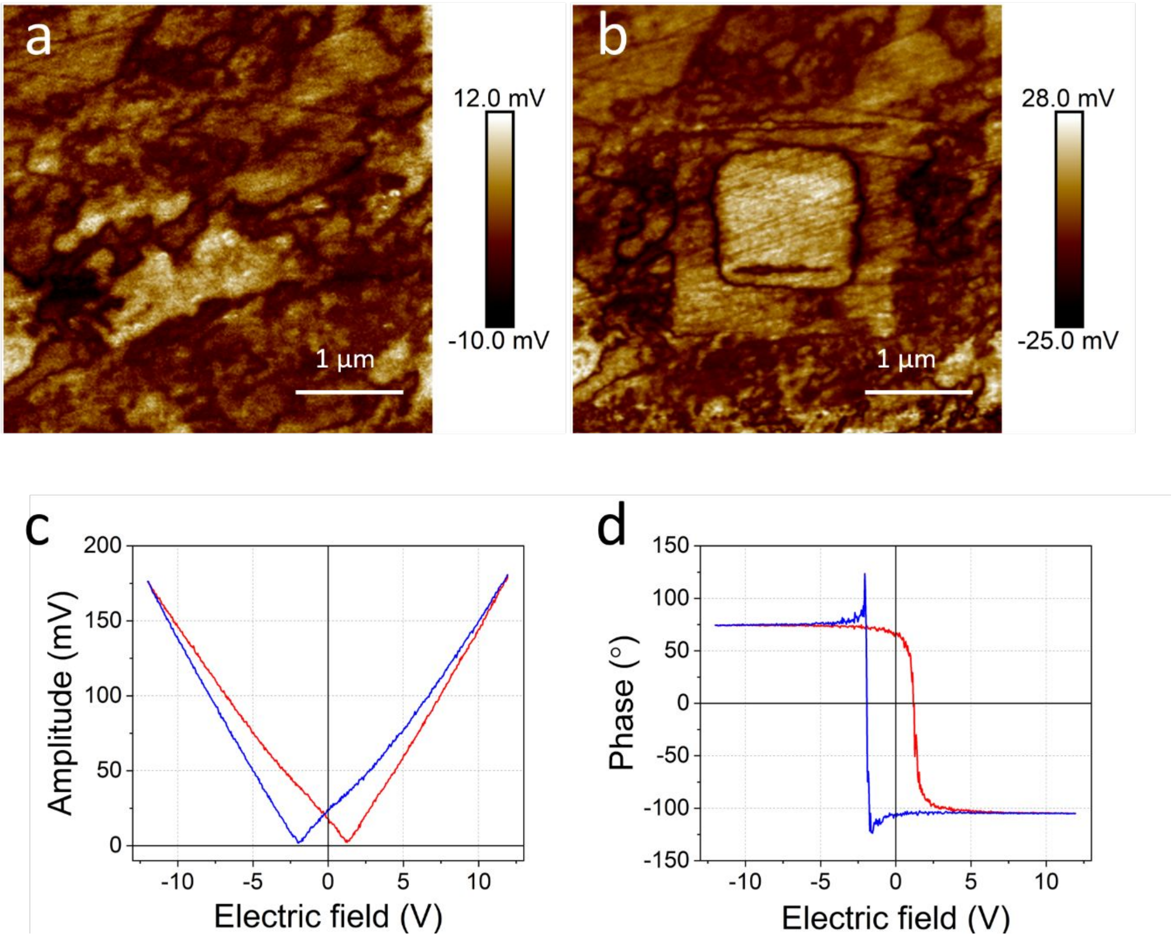

Figure S2. Piezoresponse force microscopy (PFM) images of (a) unpoled and (b) poled BST246 ceramic samples showing (a, b) magnitude, (c) amplitude-electric field and (d) phase-electric field loops.

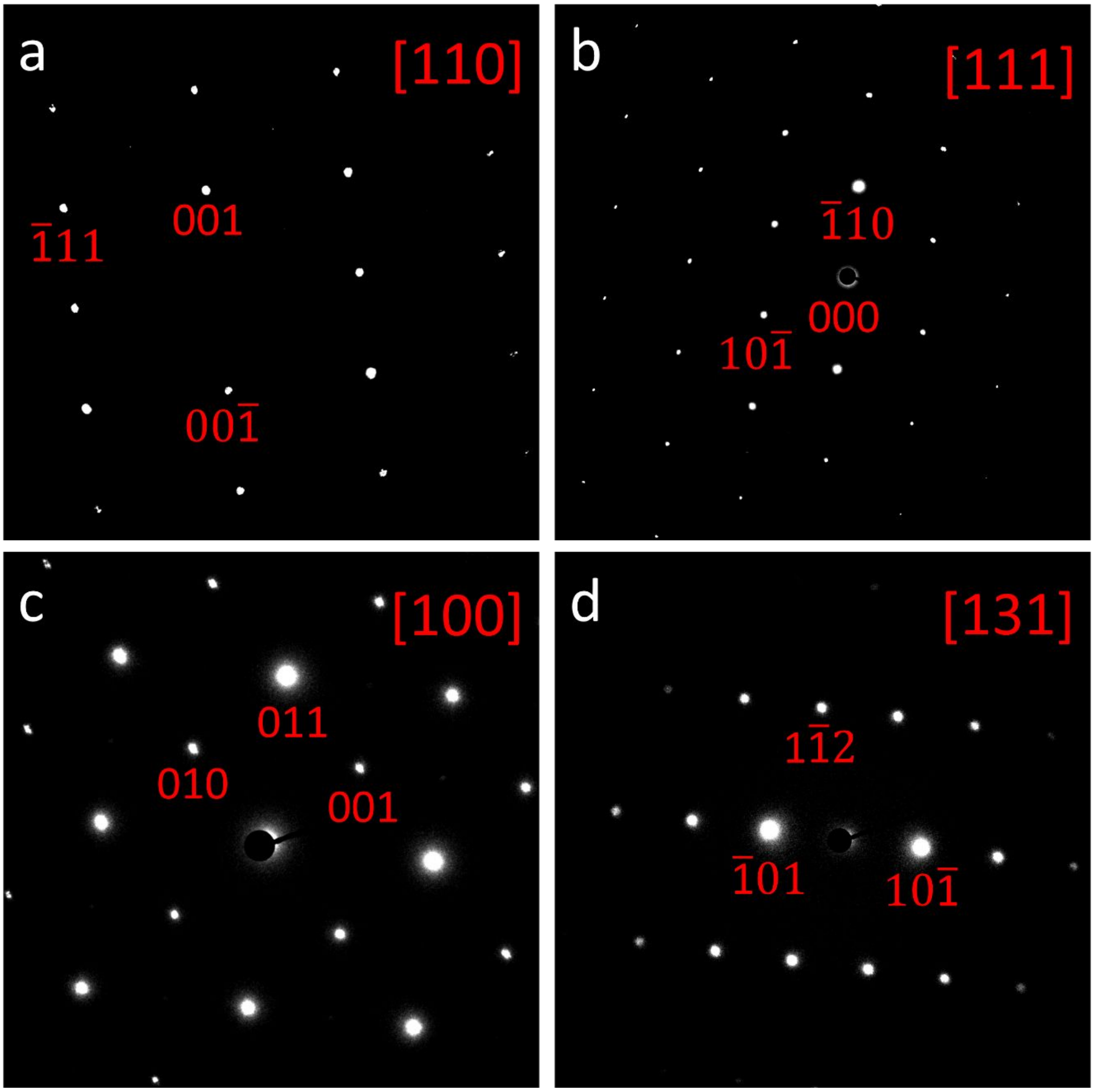

Figure S3. Selected area electron diffraction (SAED) images of (a, b) unpoled and (c, d) poled BST246 in different crystallographic directions.

Table S1. Crystal and refinement parameters for BST246 at (a) room temperature (b) 150 °C and (c) 400 °C based on fits to high-resolution neutron diffraction data. Estimated standard deviations are given in parentheses.

**(a)**		
**$Ba_{0.08}Sr_{0.12}Bi_{0.4}Na_{0.4}TiO_3$ @ 25 °C**	*Pm*-3*m* model	*P*4*mm* model
**Weight Fraction**	50.14%	49.86%
**Unit cell dimensions**	*a* = 3.91464(1) Å	*a* = 3.89988(2) Å<br>*c* = 3.95166(5) Å
**Volume (Å^3)**	59.990 (1)	60.101 (1)
**Z**	1	1
**D_{calc} (g cm^{-3})**	5.818	5.807
**R-factors[a]**	R_{wp} = 0.0517<br>R_p = 0.0492<br>R_{ex} = 0.0149<br>R_{F^2} = 0.4518	
**No. of variables**	30	
**No. of profile points**	6511	

**(b)**		
**$Ba_{0.08}Sr_{0.12}Bi_{0.4}Na_{0.4}TiO_3$ @ 150 °C**	*Pm*-3*m* model	*P*4*mm* model
**Weight Fraction**	91.29%	8.71%
**Unit cell dimensions**	*a* = 3.918873(5) Å	*a* = 3.9108(2) Å<br>*c* = 3.9449(3) Å
**Volume (Å^3)**	60.184(0)	60.335(7)
**Z**	1	1
**D_{calc} (g cm^{-3})**	5.799	5.785
**R-factors[a]**	R_{wp} = 0.0354<br>R_p = 0.0365<br>R_{ex} = 0.0153<br>R_{F^2} = 0.6980	
**No. of variables**	30	
**No. of profile points**	6511	

**(c)**	
**$Ba_{0.08}Sr_{0.12}Bi_{0.4}Na_{0.4}TiO_3$ @ 400 °C**	*Pm*-3*m* model
**Unit cell dimensions**	*a* = 3.927057(3) Å
**Volume (Å^3)**	60.562(0)
**Z**	1
**D_{calc} (g cm^{-3})**	5.763
**R-factors[a]**	R_{wp} = 0.0283<br>R_p = 0.0294<br>R_{ex} = 0.0153<br>R_F^2 = 0.6909
**No. of variables**	13
**No. of profile points**	7714

[a]For definition of R-factors see reference 1.

Table S2. Refined structural parameters for BTS246 at room temperature based on fits to high-resolution neutron diffraction data using *Pm*-3*m* and *P*4*mm* models. Estimated standard deviations are given in parentheses.

**$Ba_{0.08}Sr_{0.12}Bi_{0.4}Na_{0.4}TiO_3$ @ 25 °C**						*Pm*-3*m*					
**Atom**	**Site**	***x***	***y***	***z***	**Occ.**	**U_{11} (Å^2)**	**U_{22} (Å^2)**	**U_{33} (Å^2)**	**U_{12} (Å^2)**	**U_{13} (Å^2)**	**U_{23} (Å^2)**
Ba/Sr/Bi/Na	1a	0.0	0.0	0.0	0.08/0.12/0.4/0.4	0.0669(9)	0.0669(9)	0.0669(9)	0.0	0.0	0.0
Ti	1b	0.5	0.5	0.5	1.0	0.0244(7)	0.0244(7)	0.0244(7)	0.0	0.0	0.0
O	3c	0.5	0.5	0.0	1.0	0.0396(6)	0.0396(6)	0.0126(6)	0.0	0.0	0.0
						*P*4*mm*					
**Atom**	**Site**	***x***	***y***	***z***	**Occ.**	**U_{11}(Å^2)**	**U_{22} (Å^2)**	**U_{33} (Å^2)**	**U_{12} (Å^2)**	**U_{13} (Å^2)**	**U_{23} (Å^2)**
Ba/Sr/Bi/Na	1a	0.0	0.0	-0.051(1)	0.08/0.12/0.4/0.4	0.0304(8)	0.0304(8)	0.024(1)	0.0	0.0	0.0
Ti	1b	0.5	0.5	0.5	1.00	0.0087(7)	0.0087(7)	0.007(2)	0.0	0.0	0.0
O1	1b	0.5	0.5	0.022(1)	1.00	0.032(1)	0.032(1)	0.048(3)	0.0	0.0	0.0
O2	2c	0.0	0.5	0.442(1)	1.00	0.0095(7)	0.024(1)	0.032(1)	0.0	0.0	0.0

**$Ba_{0.08}Sr_{0.12}Bi_{0.4}Na_{0.4}TiO_3$ @ 150 °C**						*Pm*-3*m*					
**Atom**	**Site**	***x***	***y***	***z***	**Occ.**	**U_{11} (Å^2)**	**U_{22} (Å^2)**	**U_{33} (Å^2)**	**U_{12} (Å^2)**	**U_{13} (Å^2)**	**U_{23} (Å^2)**
Ba/Sr/Bi/Na	1a	0.0	0.0	0.0	0.08/0.12/0.4/0.4	0.0645(3)	0.0645(3)	0.0645(3)	0.0	0.0	0.0
Ti	1b	0.5	0.5	0.5	1.0	0.0210(3)	0.0210(3)	0.0210(3)	0.0	0.0	0.0
O	3c	0.5	0.5	0.0	1.0	0.0382(2)	0.0382(2)	0.0118(2)	0.0	0.0	0.0
						*P*4*mm*					
**Atom**	**Site**	***x***	***y***	***z***	**Occ.**	**U_{11} (Å^2)**	**U_{22} (Å^2)**	**U_{33} (Å^2)**	**U_{12} (Å^2)**	**U_{13} (Å^2)**	**U_{23} (Å^2)**
Ba/Sr/Bi/Na	1a	0.0	0.0	-0.037(3)	0.08/0.12/0.4/0.4	0.012(1)	0.012(1)	0.021(4)	0.0	0.0	0.0
Ti	1b	0.5	0.5	0.5	1.00	0.004(2)	0.004(2)	-0.004(1)	0.0	0.0	0.0
O1	1b	0.5	0.5	0.070(3)	1.00	0.14(1)	0.14(1)	0.025(8)	0.0	0.0	0.0
O2	2c	0.0	0.5	0.436(2)	1.00	-0.002(1)	0.008(2)	0.024(2)	0.0	0.0	0.0

**$Ba_{0.08}Sr_{0.12}Bi_{0.4}Na_{0.4}TiO_3$ @ 400°C**						*Pm*-3*m*					
**Atom**	**Site**	***x***	***y***	***z***	**Occ.**	**U_{11} (Å^2)**	**U_{22} (Å^2)**	**U_{33} (Å^2)**	**U_{12} (Å^2)**	**U_{13} (Å^2)**	**U_{23} (Å^2)**
Ba/Sr/Bi/Na	1a	0.0	0.0	0.0	0.08/0.12/0.4/0.4	0.0681(2)	0.0645(3)	0.0645(3)	0.0	0.0	0.0
Ti	1b	0.5	0.5	0.5	1.0	0.0222(3)	0.0210(3)	0.0210(3)	0.0	0.0	0.0
O	3c	0.5	0.5	0.0	1.0	0.0411(2)	0.0411(2)	0.0118(1)	0.0	0.0	0.0

Table S3. Selected bond lengths and angles in the tetragonal phase of BST246. Estimated standard deviations are given in parentheses.

P4mm	**Ti-O1 Bond length (Å)**	**Ti-O1 Bond length (Å)**	**Ti-O2 Bond length (Å)**	**O2-Ti-O2 Angle (°)**	**O1-Ti-O2 Angle (°)**	**M-O1-M [a] Angle (°)**
**25 °C**	1.889(5)	2.063(5)	1.9632(5)	166.69(2)	83.35(1)	168.02(1)
**100 °C**	1.870(7)	2.076(7)	1.9662(6)	166.67(3)	83.34(1)	167.72(2)
**110 °C**	1.853(8)	2.093(8)	1.9693(8)	165.3(4)	82.67(1)	166.61(3)
**120 °C**	1.820(9)	2.125(9)	1.970(1)	165.1(5)	82.57(2)	165.9(4)
**130 °C**	1.78(1)	2.16(1)	1.971(1)	165.0(6)	82.52(2)	164.6(5)
**140 °C**	1.73(1)	2.21(1)	1.971(1)	165.1(6)	82.54(3)	163.2(6)
**150 °C**	1.69(1)	2.24(1)	1.971(1)	165.3(6)	82.67(3)	162.5(7)

[a]M = A-site cations.

Reference

1. A.C. Larson, R.B. Dreele, (1987) *Los Alamos National Laboratory Report*. No. LAUR-86-748.
